# Biology of human milk oligosaccharides: From basic science to clinical evidence

**DOI:** 10.1111/jhn.12990

**Published:** 2022-02-02

**Authors:** Norbert Sprenger, Hanne L. P. Tytgat, Aristea Binia, Sean Austin, Atul Singhal

**Affiliations:** ^1^ Nestlé Institute of Health Sciences Nestlé Research, Société des Produits Nestlé S.A. Lausanne Switzerland; ^2^ Nestlé Institute of Food Safety and Analytical Sciences Nestlé Research, Société des Produits Nestlé S.A. Lausanne Switzerland; ^3^ Institute of Child Health University College London London UK

**Keywords:** animal milk, development, growth, human milk, immunity, infections, microbiota

## Abstract

Human milk oligosaccharides (HMOs) have been researched by scientists for over 100 years, driven by the substantial evidence for the nutritional and health benefits of mother's milk. Yet research has truly bloomed during the last decade, thanks to progress in biotechnology, which has allowed the production of large amounts of bona fide HMOs. The availability of HMOs has been particularly crucial for the renewed interest in HMO research because of the low abundance or even absence of HMOs in farmed animal milk. This interest is reflected in the increasing number of original research publications and reviews on HMOs. Here, we provide an overview and critical discussion on structure–function relations of HMOs that highlight why they are such interesting and important components of human milk. Clinical observations in breastfed infants backed by basic research from animal models provide guidance as to what physiological roles for HMOs are to be expected. From an evidence‐based nutrition viewpoint, we discuss the current data supporting the clinical relevance of specific HMOs based on randomised placebo‐controlled clinical intervention trials in formula‐fed infants.

## Key points

- This review discusses different aspects of human milk oligosaccharides (HMOs): from their chemistry to biology.

- It provides a comprehensive overview of our current understanding of the clinical relevance of HMOs for infant development and health.

- Cumulative evidence from clinical observations and interventions backed by mechanistic basic research data indicates that HMOs are a meaningful and important component of human milk.

- Increasing evidence suggests that specific HMOs help establish immune competence, both local and systemically, partly through their effect on the metabolite activity of specific microbes mainly *Bifidobacterium* species.

- HMOs may also participate in a gut–brain connection, thereby modulating brain and cognitive development.

- HMOs likely act in concert with other bioactive components and act via different mechanisms that converge to specific functions.

## Graphical abstract

An illustration of the human milk oligosaccharide (HMO) mediated microbiome–host interaction, highlighting its importance for the biology of HMOs for infant health.

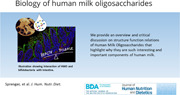

## INTRODUCTION

Human milk is the natural, adapted and sole recommended nutrition for infants. It provides not only nutrition for the growth of an infant, but also numerous bioactive components supporting age‐appropriate development and immune protection. Consequently, pediatric societies and the World Health Organization (WHO) recommend exclusive breastfeeding to 6 months of age and breast‐feeding to be continued to at least 2 years of age.^1^, ^2^ Breast milk is mostly composed of water and various solid components with nutritive values and bioactive functions.

Among the breast milk bioactive components, human milk oligosaccharides (HMOs) are a highly represented category, both in terms of amounts and structural diversity. Together with their resemblance to mucous and mucosal surface glycans, and the fact that they are largely undigestible, this has triggered extensive basic and applied research. To date, more than 160 different HMO structures have been described^3^, ^4^ with an estimated total concentration ranging between 5 and 15 g L^–1^.^5^, ^6^, ^7^

Historically, HMOs were mainly recognised as a fraction in breast milk related to the presence of beneficial bacteria such as bifidobacteria and lactobacilli in infant feces.^8^ However, there is now increasing evidence that HMOs could also contribute to broader health benefits of human milk. For over a century, breast milk has been recognised as protecting infants from morbidity and mortality. Indeed, breastfed infants generally experience less gastrointestinal and respiratory infectious illnesses, and show higher cognitive development and lower risk for being overweight or obese.^9^ The potential effect of breastfeeding on development of allergies later in life is less clear. Notably, in infants born preterm, breast milk reduces the risk of life‐threatening necrotising enterocolitis (NEC), as well as the risk of late onset sepsis, and supports medical treatments to prevent bronchopulmonary dysplasia.^10^, ^11^, ^12^ These benefits of human milk for the infant are key inspirations in the quest to understand the physiological roles of breast milk components such as HMOs.

As Ajit Varki wrote; ‘Nothing in Glycobiology Makes Sense, except in the Light of Evolution’.^13^ This can serve as a guiding principle when trying to understand the variety and abundance of HMOs found in breast milk and their variability between mothers. In this work, we discuss different aspects of HMOs: from their chemistry to biology, aiming to provide an overview of our current understanding on their clinical relevance for infant development and health.

## WHAT ARE HMOS?

HMOs are non‐lactose oligosaccharides found in human milk. A stricter definition may be that HMOs are not only present, but also produced directly by the lactating mother's mammary glands. From a physiological angle, HMOs are not digested in the infant gut and hence are not part of the nutritive breast milk components, but, as a result of their numerous roles, are considered bioactive constituents.

From a chemical perspective, all known HMOs are elongations of the milk sugar lactose with one or several of the following monosaccharides: galactose (Gal), *N*‐acetyl‐glucosamine (GlcNAc), *N*‐acetyl‐galactosamine (GalNAc), fucose (Fuc) and sialic acid (*N*‐acetyl‐neuraminic acid [NeuAc]). Lactose is formed solely in the lactating mammary glands by the lactose synthase complex, starting from uridine diphosphate galactose (UDP‐Gal) and glucose (Glc). Lactose synthase is a heterodimer composed of the milk protein alpha‐lactalbumin and the enzyme beta‐1,4‐galactosyltransferase 1, which is encoded by the *B4GALT1* gene. The *B4GALT1* gene codes for two enzymatic forms that result from two distinct transcription initiation sites and subsequent post‐translational processing. The ubiquitously present first form, a type II membrane‐bound, trans‐Golgi resident protein, is involved in glycoconjugate biosynthesis adding Gal from UDP‐Gal to GlcNAc. The second transcription product results in the soluble lactose synthase, producing lactose by adding Gal from UDP‐Gal to free Glc.

Lactose synthase is not reported to further elongate lactose with additional Gal. Rather, the next step of lactose elongation is brought about by a series of other glycosyltransferases (GTs) with different specificities as recently reviewed.^14^ Lactose can be elongated by the disaccharides lacto‐*N*‐biose (LNB; β‐Gal‐(1→3)‐β‐GlcNAc) and *N*‐acetyllactosamine (LacNAc; β‐Gal(1→4)‐β‐GlcNAc) resulting in the tetrasaccharides lacto‐*N*‐tetraose (LNT) and lacto‐*N*‐*neo*tetraose (LNnT), respectively. The exact pathway leading towards the production of the latter two HMOs is yet to be discovered. Both LNT and LNnT are also further elongated by additional LNB and LacNAc units. Although the involved enzymes are not described, it is likely by tight sequential addition of GlcNAc and Gal units. The intermediate lacto‐*N*‐triose with only a GlcNAc added to lactose is only rarely reported in human milk.^15^, ^16^ Both LNT and LNnT as well as their further LNB‐ and LacNAc‐elongated descendants can be decorated with Fuc and NeuAc. Because many GTs are involved in the formation of several linkage types, a large variety of structures differing in composition, conformation and chain length is found in human milk.^3^, ^14^, ^17^ Trace amounts of galactosyl‐lactoses (GLs), mainly 6'‐GL, are also found in human milk^7^, ^18^, ^19^ and, although the enzymes involved in their formation are not known, interestingly, no further elongation with Fuc or NeuAc has been reported. This could indicate that the GL synthesis is not localised in the endomembrane system together with the GTs that are involved in the major HMO synthesis pathways. We speculate that different microbes present in milk, albeit in low concentration, could be responsible for synthesising these GLs in breast milk, similar to the GL formation during milk fermentation with starter cultures.^20^

**Figure 1:**
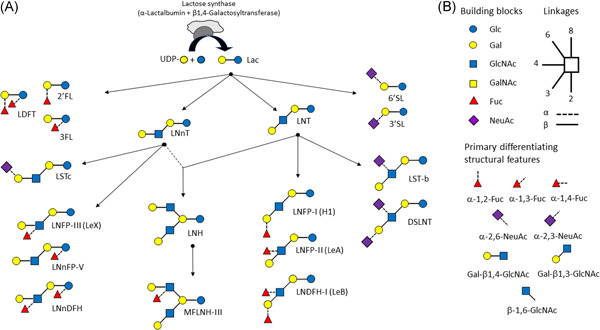
Main human milk oligosaccharides (HMOs) found in human milk. (a) Illustration of main HMOs and their synthetic route starting from lactose that is formed by lactose synthase complex from UDP‐Gal and Glc in the mammary glands. (b) Key to the monosaccharide building blocks and linkages of HMOs and illustration of the eight primary differentiating structural features represented in HMOs. 2'FL, 2'Fucosyllactose; 3FL, 3Fucosyllactose; 3'SL, 3'Sialyllactose; 6'SL, 6'Sialylactose; DSLNT, DisialyllactoNtetraose; Fuc, fucose; Gal, galactose; GalNAc, *N*‐acetyl‐galactosamine; GlcNAc, *N*‐acetyl‐glucosamine; Glc, glucose; NeuAc, *N*‐acetylneuraminic acid (sialic acid); LDFT, Lactodifucosyltetraose; Le (A,B,X), Lewis (A,B,X); LNnT, LactoNneotetraose; LNT, LactoNtetraose; LNFP (I,II,III), LactoNfucopentaose (I,II,III); LNnFP V, LactoNneofucopentaose V; LNnDFH, LactoNneodifucosylhexaose; LNDFH I, LactoNdifucosylhexaose I; LST (b,c), SialyllactoNtetraose (b,c); MFLNH III, MonofucosyllactoNhexaose III

Elongation of core HMOs with Fuc is catalysed by fucosyltransferases (FUT), which add Fuc from guanosine diphosphate fucose (GDP‐Fuc) to lactose (or other acceptor oligosaccharides) through different linkages. These are encoded by *FUT2* and *FUT3*, respectively called the secretor and Lewis gene, as well as further *FUT* genes (e.g., FUT5,6). This leads for example to the formation of 2'‐fucosyllactose (2'‐FL), 3‐fucosyllactose (3‐FL) and lacto‐difucosyltetraose (LDFT; 2',3‐difucosyllactose) (Figure 1). Similarly, several sialyltransferases such as St3Gal4 and St6Gal1 add NeuAc (*N*‐acetylneuraminic acid) to lactose (or other acceptor oligosaccharides) in different linkages, resulting in for example 3'‐sialyllactose (3'‐SL) and 6'‐sialyllactose (6'‐SL). The formation of the main HMOs that are generally analysed in most studies is depicted in Figure 1.

Based on their chemical composition HMOs may be classified into different categories. The most apparent subclassification is the separation of acidic HMOs harboring one or more sialic acids from neutral HMOs. The latter can be further grouped into those containing one or more fucose units vs. those without a fucose moiety. The acidic sialylated HMOs may be further split to discern acidic non‐fucosylated and acidic fucosylated HMOs.

Biology is not only directed by chemical composition, but, importantly, also by the three‐dimensional structure. Hence, it is essential to consider structural features when classifying HMOs. The same sugar monomer can be attached in different ways by different enzymes to another saccharide unit. For example, adding a Fuc to Lac can result both in 2'FL or in 3‐FL depending on the catalysing GT. Investigating the structures of the major reported HMOs, eight primary differentiating structural features can be distinguished (Figure 1b). Generally, and as recently reviewed, each of these structural features is formed by a specific GT, although some can be formed by several GTs.^14^ Although FUT2 specifically adds fucose in α1,2 to an acceptor galactose, FUT3 forms both α1,4 and α1,3 linkages between fucose and GlcNAc, as well as Glc. However, an additional FUT can also form the α1,3 linkages between Fuc and Glc (Figure 1).^21^

**Figure 2:**
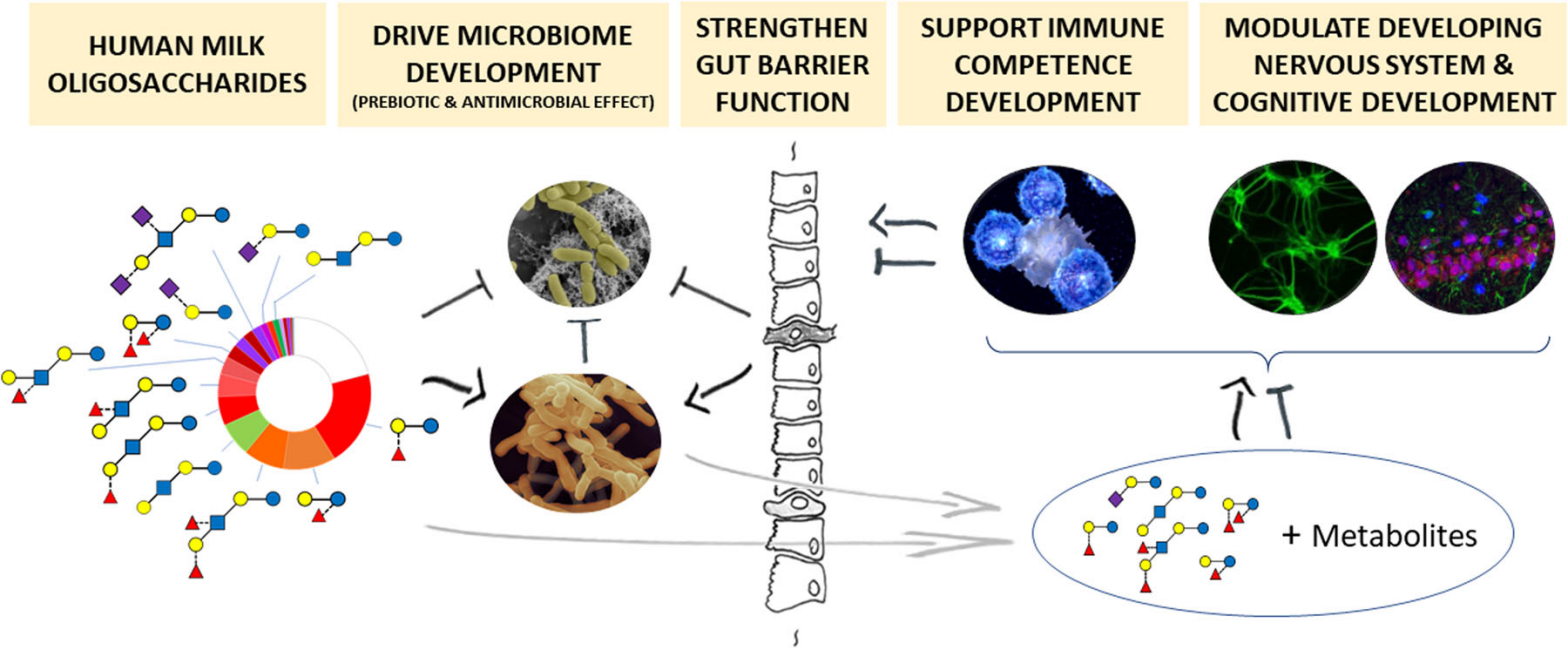
Summary illustration of main expected human milk oligosaccharide (HMO) functions reported in literature

The presence or absence of the FUT2 and FUT3 enzymes also allows further stratification of human milk. The FUT2 genotype is linked to secretor status (Se), with women of a *FUT2* ^*−/−*^ genotype being unable to produce α1,2 fucosylated HMOs, such as 2'‐FL and LNFP‐I. The FUT3 enzyme determines the Lewis type (Le), resulting in strong reduction of α1,3 and absence of α1,4 fucosylated HMOs in milk. Lewis and Secretor type thus allow grouping of human milk in four main milk groups, also sometimes named ‘lactotypes’, which will be discussed later. Milk groups show significant differences in HMO profiles even beyond those HMOs directly affected by the *FUT2* or *FUT3* genotype.^7^ Possible explanations are alterations in acceptor (e.g., LNT) or altered donor availabilities (GDP‐Fuc) if one or the other enzyme is not active: for example, absence of a downstream fucosylation enzyme results in a higher proportion of sialylated HMO structures. HMO profiles are thus not solely dependent on the direct control by specific enzymes and their expression pattern. This means that the HMO structural diversity and composition are closely tied to their biosynthesis and any maternal factors, such as genetic background, health condition, environmental factors and diet, modulating the expression and function of maternal GTs, as well as substrate availabilities. Whether or not reported differences have a physiological significance for the suckling newborns is a topic of intensive research.

Of note, GTs involved in HMO biosynthesis in the mammary glands, such as FUT2 for example, are also expressed in other body parts, where they are involved in cell and mucous glycosylation. Therefore, HMOs may be considered as soluble lactose‐bound analogues of typical mammalian cell glycocalyx and mucous glycans, which represent a dense glycan matrix at the interface with other cells and the environment, including the microbiome.

## WHERE ARE HMOS FOUND?

Although human milk is particularly rich in both amount and number of oligosaccharides with a high diversity of structural features not generally seen in other mammals, all mammals produce the milk sugar lactose in their milk and all mammals are equipped with a series of GTs such as those involved in HMO synthesis (see earlier). Milk of different mammalian species greatly varies in amount, number and diversity of structural features of their milk oligosaccharides.^22^ Some are common to those found in human milk, whereas others are not. Among the HMOs more universally found in mammals, including monotremes (e.g., platypus), marsupials and eutherians (i.e., placental mammals), are the sialyllactoses, primarily 3'‐SL.^22^, ^23^ The milk of the egg‐laying platypus contains 3'‐SL and larger sialylated structures together with predominantly fucosylated lactose, such as LDFT, but also larger fucosylated oligosaccharides built on LNnT and LNT.^23^ Similar oligosaccharides are also reported in milk of *Echidna*, another egg‐laying mammalian species.^24^ These observations suggest that specific oligosaccharides are ancestral features of milk. As a result of the immaturity of monotremes at birth, these oligosaccharides were also suggested to be of particular importance for development and immune protection. Noteworthy, 3'‐SL and 6'‐SL are found in mouse and rat milk as highly predominant milk oligosaccharides, rendering these animals, who are born relatively immature, relevant models to study their role for growth and development.^25^, ^26^, ^27^, ^28^

Farmed animal milks also contain oligosaccharides, but at relatively low concentrations. Among them are 3'‐SL, 6'‐SL and primarily other neutral non‐fucosylated oligosaccharides such as galactosyllactoses.^29^ Generally, their concentration decreases very rapidly from colostrum to mature milk.^29^ In bovine milk, 3'‐SL is the most prominent oligosaccharide with a concentration reported to range between 50 and 100 mg L^–1^ compared to an approximately two‐fold higher amount observed in mother's milk. Notably, 3'‐SL was reported to increase in bovine milk around 2 weeks before parturition from around 100 to 700 mg L^–1^ and to steeply drop from around 800 mg L^–1^ in colostrum to around 100 mg L^–1^ by 3 days postpartum.^30^, ^31^

Several structural features characterise human as opposed to animal milk oligosaccharides. Human milk shows a predominance of type 1 structures (LNB), built on LNT cores, whereas animal milks that have larger oligosaccharides mainly show type two structures (LacNAc), built on LNnT cores.^32^, ^33^ Because humans cannot synthesise the *N*‐glycolyneuraminic acid (NeuGc) form of sialic acid, mother's milk only contains oligosaccharides with NeuAc, whereas animals do show also NeuGc on milk oligosaccharides in different proportions compared to NeuAc.^34^, ^35^ For example, goat milk contains approximately 70% NeuGc and 30% NeuAc, whereas, in bovine milk, over 95% of sialic acid is NeuAc.^36^

## ARE ALL HMOS THE SAME?

As outlined in the previous section, each HMO is structurally distinct from another. However, some commonalities exist in composition and the primary differentiating structural features. Interestingly, it should be noted that, of all theoretically possible structures, only a limited and a finite number of structures are made and present in human milk. Although HMO diversity and richness suggest many structure‐specific functions, the many different structures may also indicate a certain functional redundancy. The more universally present milk oligosaccharides, such as the sialyllactoses, are likely to contribute to similar more universal physiological needs of different mammals during the early postnatal life. On the other hand, different mammals have different postnatal nutritional and functional requirements that strongly depend on their maturity at birth, their speed of postnatal development and their environment. Hence, general milk composition differs among mammalian species and probably represents adaptation to their newborn's requirements. The question is: are milk oligosaccharides part of such an adaptation of milk?

In humans, HMO profiles strongly change by maternal polymorphisms in the Lewis blood group system (i.e. FUT2 and FUT3 polymorphisms) and by duration of lactation.^7^, ^37^, ^38^ The distribution of the Lewis blood group polymorphisms indicates that this trait strongly depends on evolutionary pressure and selection with approximately 10%–35% FUT2 (secretor) negative genotypes in different geographies.^39^, ^40^, ^41^ Population genetic studies confirm the presence of balancing selection acting upon FUT2, an indication of advantages linked to maintaining genetic variation.^39^, ^42^ The prevalence of FUT2 negative genotypes varies across different geographies, thus contributing to reported geographic differences in HMO profiles.^43^ Additional environmental and maternal factors may also contribute to HMO variability, although their effect size may be rather modest.^7^, ^43^ Comparison of HMO profiles from mothers who gave birth to preterm vs. term infants indicated that sialyllactose is slightly higher and FUT2 dependent HMOs such as 2'‐FL and LNFP I slightly lower in early milk of mothers who gave birth to a preterm infant.^18^ Whether such observations represent an adaptation of the milk for the physiological needs of the infant or rather reflect the physiological state of the mother needs further investigation. Interestingly, birth appears to trigger a program that determines how the HMO profile changes over the period of lactation.^18^ The stage of lactation is a key parameter affecting HMO profiles, with most HMOs decreasing and a few increasing in concentration over the first few months and even beyond the first year of lactation.^7^, ^38^ 3‐FL is found among the HMOs generally increasing in concentration and, although the reason for this is unknown to date, we speculate that 3‐FL may have a particular role beyond the exclusive breastfeeding period. When estimating the daily average intake, most HMOs show a relatively constant intake, whereas 3‐FL intake increases with time of lactation.^44^

In basic research models, both redundancy and specificity with selected HMOs is reported. In animal models coupled with cell‐based models and modelling, both 2'‐FL and 6'‐SL were shown to affect NEC via Toll like receptor 4 (TLR‐4) mediated route.^45^ Similarly, 2'‐FL and 3‐FL were shown to interact with dendritic cell‐specific intercellular adhesion molecule‐3‐grabbing non‐integrin (DC‐SIGN), whereas LNT did not bind.^46^ In yet another study, 6'‐SL and LNT both activated the G‐protein coupled receptor GPR35, with both together activating stronger, whereas 2'‐FL, LNnT and 3‐'SL did not activate this receptor.^47^ When it comes to stimulation of specific *Bifidobacterium* species, many more examples of redundancy and specificity exist. Such redundancies indicate their physiological importance and are likely to come into play to compensate FUT2 and FUT3 polymorphisms. Detailed observational studies coupled with mechanistic insight are warranted to understand such initial observations and hypothesis.

## MANUFACTURED HMOS ADDED TO INFANT FORMULA: ARE THEY SAFE AND WHAT ARE THEIR BENEFITS?

The relatively large amounts of diverse HMOs found in human milk contrasts with milk from farmed animals used for human nutrition, which contain only very small amounts of oligosaccharides. To close this gap for animal milk‐derived breast milk substitutes in child nutrition, different technological strategies are possible. For example, the animal milk oligosaccharide‐rich fraction can be concentrated, an approach that can provide some oligosaccharides such as the sialyllactoses but, as a result of the low initial amounts, this is technically challenging.^48^ Alternatively, individual HMOs may be produced by chemical, enzymatic or biotechnological means. Recent progress in these fields has enabled industrial production of individual HMOs that are not typically found in farmed animal milks.

Today different biotechnological processes using bacterial fermentation are the most industrially viable technologies. A handful of individual HMOs are available at industrial scale, representing some of the most abundant HMOs found in breast milk. Although they have the primary structural features of HMOs (Figure 1), they are still relatively simple compared to the some of the larger HMOs found in human milk.

These technologies involve novel processes, as defined by the regulatory authorities such as the European Food Safety Agency (EFSA) or the Federal Drug Administration (FDA). Today, a select number of HMOs (2'‐FL, 3‐FL, LDFT, LNnT, LNT, 3'‐SL, 6'‐SL) have obtained Generally Recognized As Safe (GRAS) and Novel Food status. These HMOs, produced by biotechnology, are identical in structure to those naturally present in breast milk and are therefore dubbed ‘human identical milk oligosaccharides’ abbreviated as HiMOs. These ingredients are diligently analysed, characterised and subjected to a series of safety/toxicity tests according to guidelines established by the Organisation for Economic Cooperation and Development (OECD). Generally, to best match the target application in early life nutrition, the *in vivo* OECD testing protocol with animals was adapted to include a juvenile period.^49^, ^50^

## WHAT ARE THE PHYSIOLOGICAL ROLES OF HMOS?

The physical and physiological development of infants is intricately linked with their environment, including key influences such as nutrition and the developing gut microbiome. In terms of early life nutrition, breast milk feeding with its high abundance and variable amounts of different HMOs is the optimal nutrition for the developing infant and its microbiome. Yet some HMOs may have redundant roles and some may have synergistic functional effects, and hence simple associations between particular HMOs and their health effects may be difficult to establish. Ultimately, hypotheses need to be tested and causality established through mode‐of‐action research. In the following, we provide an overview of observations from different studies with breastfed infants and intervention trials with HMO formula‐fed infants. Where possible, we discuss basic research data relevant to support clinical observations. Figure 2 illustrates the main proposed functions of HMOs.

### HMOs and the development of the early life gut microbiome

A bona fide gut colonisation with microbes starts with maternal and environmental microbiota seeding, followed in part by appropriate selection through undigested dietary components. To this end, breast milk provides a multitude of diverse oligosaccharides (HMOs), together with numerous other key components such as immunoglobulins, lactoferrin and beyond. Primarily during the recommended exclusive breastfeeding period through 4–6 months of age, infants who are exclusively or partially breastfed show a different maturation trajectory of their gut microbiome compared to their non‐breastfed peers. This is first reflected in a lower microbiota diversity index and secondly in a higher microbiota age or maturity compared to the infant's chronological age in formula fed infants.^51^, ^52^, ^53^ These measures indicate that low or no breast milk intake accelerates maturation of the gut microbiome towards that observed in adults.^52^

At the microbiota taxonomic level, breastfed infants show primarily higher relative abundance of Bifidobacteriaceae family members during the early exclusive breastfeeding period. Notably, primarily strains from the classes Actinobacteria, including Bifidobacteriaceae, and Bacteroidia were found to be vertically transferred from the mother to her infant.^54^ Additionally, breast milk was identified as the most important covariate explaining microbiome variance at genus and species taxonomy levels, as well as the microbiome functional capacity.^53^ The importance of bifidobacteria especially during early life for gut ecology and development is well established^55^, ^56^, ^57^ and highlights the importance of maternal seeding and feeding.

Generally, HMOs are not digested by the infant's digestive enzymes, although intestinal neuraminidase cleaving sialylated HMOs may be an exception.^58^ Different studies with breastfed infant–mother dyads have investigated HMO profiles in breast milk and infant stools. Although some studies report only relatively small changes, others found that HMO profiles in some infant stools changed more dramatically.^44^, ^59^, ^60^, ^61^ In addition to important inter‐individual variability, HMO profiles are strongly associated with specific microbiota taxa, primarily *Bifidobacterium*, *Bacteroides* and *Lactobacillus*.^61^

*Bifidobacterium*species are genetically particularly well equipped to take advantage of the HMO diversity and abundance in breast milk.^62^, ^63^, ^64^ Their dominance in early life is largely attributed to this unique genetic glycan‐foraging capacity, which is likely a result of coevolution with the host.^65^, ^66^, ^67^ Several studies have investigated the complex glycan degradation capabilities of early life colonisers and all showed the unique capacity of bifidobacteria to utilise HMOs, whereas other strains favor less complex oligosaccharides and sugar monomers as carbon sources for growth.^68^, ^69^, ^70^ In general, two large strategies are deployed by bifidobacteria to utilise HMOs: (1) the secretion of glycosidases that externally degrade HMOs followed by the uptake of sugar monomers by the bifidobacteria (e.g., *Bifidobacterium bifidum* and some *Bifidobacterium longum* strains) and (2) the expression of dedicated HMO transporters that can internalise HMOs for further internal degradation.^63^, ^71^ The latter strategy is found for example in *Bifidobacterium breve*, *Bifidobacterium longum* subsp. *infantis* and most other *B. longum* strains.^63^, ^71^ Interestingly, strain‐specific differences in HMO utilisation capacity were reported within the same *B. breve* and *B. longum* subsp. *infantis* species.^63^, ^67^, ^72^, ^73^ To what extent these strain specificities reflect a personalised mother–infant exchange of microbes and nutrition, or indicate a gradual gene pool loss of key microbes, needs to be established in detail.

Another group of microbes able to utilise HMOs for growth are *Bacteroides* species.^74^ However, in a gnotobiotic mouse model associated with a *Bacteroides* and a *Bifidobacterium* species, the HMO LNnT boosted the abundance of the *Bifidobacterium* species over the *Bacteroides*, although both species were able to utilise LNnT.^75^ Although the *Bacteroides* species can equally use mucous glycans and HMOs, the *Bifidobacterium* species can only use HMOs as growth substrate and HMOs appear to provide a selective advantage to *Bifidobacterium* species. Other early gut colonisers such as the Enterobacteriaceae, which includes several pathogens, are generally not able to grow on HMOs.^76^ For *Streptococcus* species, 2'‐FL contrary to lactose or galactooligosaccharides (GOS) was shown not to allow for *Streptococcus mutans* growth and LNT was identified to interfere with a Group B *Streptococcus* (GBS) cell wall synthesis, which leads to cell death and higher sensitivity to antibiotics.^77^, ^78^, ^79^, ^80^ The former may have a clinical relevance for oral health, whereas the latter may be of relevance to reduce sepsis risks and antibiotic dosing especially in infants born preterm. A second reason behind the dominance of *Bifidobacterium* species in the gut in early life, is its social behavior. Collaboration and cross‐feeding may occur between different *Bifidobacterium* strains and between different species.^66^, ^81^, ^82^, ^83^ The first phenomenon relies on the presence of different HMO utilisation loci in different bifidobacteria, resulting in crossfeeding chains, further enhancing their dominance.^66^ Crossfeeding of other strains relies on several HMO metabolites produced by bifidobacteria, such as the short chain fatty acid acetate supporting *Anaeostipes caccae* a butyrogenic species^71^, ^84^ or sugar oligo‐ and monomers supporting growth of other species unable to degrade HMOs, such as lactobacilli.^69^ An elegant study showed that an early life gut microbiome community with a very high Bifidobacteriaceae dominance is established in presence of *Bifidobacterium* strains, in this case specific strains of *B. breve*, which have the genetic make‐up to utilise specific HMOs such as 2'‐FL.^67^ Concomitantly, stool from infants with a microbiome harboring this 2'‐FL utilising capacity was also shown to have lower pH, higher acetate and lower remaining 2'‐FL from breast milk, indicating higher metabolic activity. Such gut ecology changes with higher acetate, likely combined with other metabolites, was shown in animal models to lead to improved protection against gastrointestinal and respiratory tract infections.^85^, ^86^ Infant gut microbiome HMO utilisation capacity was recently shown to relate to less inflammatory markers and specifically *B. longum* subsp. *infantis* derived metabolites such as indolelactate were shown to drive such pathways.^87^, ^88^ As expected, such bifidobacteria activity driven processes are very relevant for appropriate immune competence development.

### HMOs and infant anthropometry

Along with environmental, genetic, epigenetic and metabolic factors, the developing gut microbiome is considered as a key factor affecting infant growth.^89^ Considerable evidence suggests that the disruption of an age‐appropriate gut microbiota assembly and succession could lead to growth faltering. For example, in infants born preterm, delayed microbiota succession or maturation was related to lower weight‐for‐age *z*‐score (WAZ), leading to the hypothesis that the microbiome, influenced by nutrition, may play a causal role in promoting growth.^90^ Similarly, studies focusing on undernourished infants found that an altered gut microbiota could be causally related to growth, with causality being established via studies in gnotobiotic animal models.^91^, ^92^, ^93^, ^94^ Similar to observations in preterm infants, the microbiota in stunted or undernourished infants is immature, as concluded from a modelling approach that used the microbiota composition to predict an infant's chronological age.^91^, ^93^ Based on their effect on the establishing gut microbiota, HMO are also investigated in relation to infant growth as summarised in Table 1.

**Table 1:** Summary of reported associations between human milk oligosaccharides (HMOs) and anthropometric measures

Anthropometry measure	Infant age (months)	Associated HMOs		Study type	Feeding mode	Reference
		Positive	Negative			
Stunted growth			Fucosyl‐ HMOs sialyl‐ HMOs	Obs	BF	^92^
Height/length‐for‐age *z*‐score change	6–12	HMOs abundance^a^		Obs	BF	^96^
Height/length‐for‐age *z*‐score	5	LNFP I + III, DFLNHa		Obs	BF	^107^
Height/length *z*‐score	3–12	2'‐FL	LNnT, LST‐b	Obs	BF	^100^
Height/length‐for‐age *z*‐score^b^	5	3'‐SL, LDFT	LNnT, DFLNH	Obs	BF	^101^
Weight	6		LNFP I	Obs	BF	^97^
Weight gain	1–6		LNFP II	Obs	BF	^104^
Weight *z*‐score	3–12	2'‐FL, 3‐FL	LNnT	Obs	BF	^100^
Weight velocity^b^	0–5	2'‐FL	LNnT	Obs	BF	^101^
Weight‐for‐age *z*‐score	2–6	3'‐SL, 6'‐SL		Obs	BF	^106^
Weight‐for‐age *z*‐score	5	3'‐SL	LST‐c	Obs	BF	^107^
Weight‐for‐length gain	0–4	3'‐SL		Obs	BF	^103^
Head circumference SDS	3–12	Non‐secretor milk		Obs	BF	^105^
BMI‐for‐age *z*‐score^b^	5		6'SL	Obs	BF	^101^
BMI SDS	3–6	Non‐secretor milk		Obs	BF	^105^
Lean mass	6		LNFP I	Obs	BF	^97^
Fat mass	6	LNFP II, DSLNT	LNFP I	Obs	BF	^97^
Fat mass	2–6	3'‐SL, 6'‐SL, DSLNT		Obs	BF	^106^
Fat mass index^b^	5	2'‐FL, LDFT,	LNnT, DFLNH	Obs	BF	^101^
Percent fat	6		LNnT	Obs	BF	^97^
Weight, length, head circumference and their *z*‐scores	0–4	No association seen in secretor positive vs. secretor negative milk		Obs	BF	^99^, ^103^
Weight, length, head circumference and their *z*‐scores	0–4	No differences observed with 2'‐FL alone or combined with LNnT or LNT, 3'‐SL, 6'‐SL, LDFT or 3‐FL		RCT	FF	^109^, ^110^, ^111^, ^113^, ^114^, ^115^, ^116^

Abbreviations: BF, breastfed; BMI, body mass index; DFLNH, DifucosyllactoNhe; FF, formula fed; HMO, human milk oligosac charides; Obs, observational; RCT, randomised placebo‐controlled trial; SDS, standard deviation score.

^a^ Abundance assessed by integration of collected ion signals.

^b^ Association seen in secretor positive milk fed infants only.

Interestingly, in two Malawian mother‐infant dyad cohorts with a total of 303 infants, lower fucosylated and sialylated HMOs (for the latter primarily LST‐b) were observed in breast milk of non‐secretor mothers whose infants were stunted compared to those showing normal growth.^92^ No significant difference was seen in secretor mothers whose child was stunted or growing normally. Using gnotobiotic mice with a microbiota from stunted infants and sialylated oligosaccharides derived from bovine milk, primarily 3'‐SL, a link between the HMOs, the microbiota and infant growth was reproduced.^92^ This indicates that specific milk oligosaccharides may act via the microbiota to modulate infant growth. Importantly, a mechanistic link between the sialylated bovine milk oligosaccharides, essentially sialyllactose and bone formation was shown using the gnotobiotic mouse model.^95^ A recent observational study in rural Malawi (*n* = 647) reported a significant association of HMO absolute abundance at 6 months with length‐for‐age change from 6 to 12 months, but no relationship between sialylated HMOs and growth.^96^

Several observational studies have investigated a possible link between breast milk oligosaccharides and infant growth in well‐nourished breastfed infants born at term. Although some associations were found, only a few are consistent across different studies.^96^, ^97^, ^98^, ^99^, ^100^, ^101^, ^102^, ^103^, ^104^, ^105^ For example, in Hispanic mother‐infant pairs (*n* = 157), a higher LNFP II concentration in breast milk at 1 month of lactation was associated with lower weight gain from 1 to 6 months of age,^104^ whereas, in a previous analysis of a small cohort (*n* = 25), LNFP II at 6 months of lactation was associated with higher fat mass at 6 months of age.^97^ However, major differences exist among the different studies with respect to design, geography, sampling during lactation, number of time points at which growth parameters are assessed, the specific HMOs that were analysed and the statistical methods applied to model the associations. Disialyllacto‐*N*‐tetraose (DSLNT) concentration at 6 months of lactation was associated with higher fat mass of infants at 6 months of age.^97^ In another US cohort, DSLNT intake at 2 months was also related to subsequent fat mass through 6 months.^106^ Additionally, in the same study, 3'‐SL and 6'‐SL at 2 months of lactation were associated with higher fat mass and WAZ from 2 to 6 months of age.^106^ In a small cohort of mothers and infants from The Gambia, 3'‐SL was found to be associated with an increase in WAZ, whereas other sialylated HMOs such as LST‐c showed the opposite association.^107^ In a recent European multicenter study of 370 mother–infant dyads, 3'‐SL was the only HMO associated with higher weight for length gain during the first 4 months of lactation.^103^ Similarly, associations between fucosylated HMOs (e.g., 2'‐FL, LNFP I, LNFP II) or neutral non‐fucosylated HMOs (e.g., LNnT) and growth, show conflicting results in different observational studies. In two studies, 2'‐FL was associated with higher growth velocity and fat mass index from birth to 5 months and length and weight *z*‐scores at 3 months of age.^100^, ^101^ In the same studies, LNnT was inversely correlated with 2'‐FL, and it was proposed that the 2'‐FL/LNnT ratio at 3 months is associated with higher length and weight *z*‐scores.^100^, ^101^ To date, no other study has confirmed these observations.

Biological plausibility for some of these observations may be built on the following hypothesis. As previously mentioned, HMO intake may increase food efficiency through microbiome related processes, explaining how specific HMO–microbiota pairs could affect infant anthropometry. Another hypothesis, proposed recently, is that HMOs affect food‐responsiveness and appetite through a microbiome driven process that affects the entero‐endocrine system or central nervous system. Indeed, specific HMOs were recently shown to be both positively and negatively associated with food‐responsiveness.^108^ However, a consistent picture explaining associations between HMOs and anthropometric findings has not yet emerged.

As a result of insufficient data, it is not possible to explain the above inconsistent and often contradictory associations between HMO and infant growth. However, we can speculate that factors such as gut microbiota differences (e.g., epigenetic and genetic differences) and maternal nutritional status during pregnancy may be important confounding variables. It should also be noted that, generally, the observed effect sizes of associations between breast milk HMOs and growth in healthy well‐nourished infants are modest and within normal growth trajectories. Moreover, because associations do not imply a causal relationship, causality needs to be established using randomised placebo‐controlled interventional trials (RCTs) and supporting mechanistic studies.

Under controlled and randomised conditions, growth of infants fed formula containing individual HMOs in different combinations, 2'‐FL either alone or in combination with LNnT or in combination with LNT, 3'‐SL, 6'‐SL, LDFT or 3‐FL, was similar to control formula‐fed infants, without HMOs and whenever assessed, equivalent to infants exclusively breastfed for at least 4 months.^109^, ^110^, ^111^, ^112^, ^113^, ^114^, ^115^, ^116^ These RCTs assessed infant growth in healthy infants born at term with growth as the primary study end point. All trials concluded that addition of specific HMO or HMO blends to infant formula are well tolerated and allow for age‐appropriate growth. However, whether the addition of specific HMOs could help to improve growth in specific conditions of malnutrition and growth faltering, or in infants born preterm, is unknown and needs to be established in RCTs coupled with mechanistic studies. Clinical findings on the link between HMOs and immunity and infections are summarised in Table 2.

**Table 2:** Summary of observed associations between human milk oligosaccharides (HMOs) and reduced risks for infant health related outcome measures

Measure	Infant age (months)	HMOs	study type	feeding mode	References
Necrotising enterocolitis	Preterm	DSLNT	Obs	HM	^127^, ^128^
Necrotising enterocolitis	Preterm	HMO diversity	Obs	HM	^129^
Immunoglobulin E‐associated eczema^a^	48	2'‐FL, secretor positive milk	Obs	BF	^136^
Cow milk protein allergy	18	LNFP III, LNFP I, 6'‐SL, DSLNT	Obs	BF	^141^
Sensitisation	12	HMO profile^b^	Obs	BF	^145^
Plasma cytokine profile^c^, ^d^	1.5	2'‐FL	RCT	FF	^147^
Diarrhea	9	2'‐Fucosyl‐HMOs	Obs	BF	^153^
*Campylobacter*diarrhea	9	2'‐FL	Obs	BF	^153^
Morbidity	4	2'‐Fucosyl‐HMOs	Obs	BF	^107^
Diarrhea	ca 11	2'‐Fucosyl‐HMOs	Obs	BF	^159^
Morbidity	3	LNFP II	Obs	BF	^160^
Prescribed antibiotic use^d^	12	2'‐FL + LNnT	RCT	FF	^109^
Lower respiratory tract infections^d^	12	2'‐FL + LNnT	RCT	FF	^109^
Overall infections^d^	1.5	2'‐FL	RCT	FF	^111^

Abbreviations: BF, breastfed; FF, formula fed; HM, human milk fed; HMO, human milk oligosaccharides; Obs, observational; RCT, randomised placebo‐controlled trial.

^a^ In C‐section born only.

^b^ Relative higher concentrations of FDSLNH, LNFPII, LNnT, LNFPI, LSTc, FLNH and lower concentarions of LNH, LNT, 2'‐FL and DSLNH.

^c^ Interleukine receptor antagonist (IL‐1ra), IL‐1a, IL‐1b, IL‐6 and tumour necrosis factor α (TNF‐αa).

^d^ Secondary exploratory outcome measures.

### HMOs and immune competence development

Development of the immune system starts *in utero* and continues with exposure to new stimuli during postnatal development. Several stimuli important for immune development stem initially from the maternal microbiome passing through the placenta to the fetus.^117^ Postnatally, although maternal microbial metabolites continue to affect the newborn (e.g., through breast milk), exposure to metabolites and components from the infant's developing intestinal microbiome is much greater and more important. HMOs are considered to affect immune system development by their major influence on the establishment of the infant gut microbiome and its metabolic activity.

Development of the immune system is particularly important in infants born preterm, who are at increased risk of numerous health problems such as NEC, sepsis and cerebral palsy, which are all at least partly linked to an inappropriate immune reaction. Human milk feeding strongly reduces the risk of developing these diseases, probably by its immune modulating effects through components with direct action and others that involve the development of the gut microbiome.^11^, ^118^, ^119^, ^120^, ^121^ Several blends and individual HMOs have been investigated for their protective effects in preclinical NEC models. In rodent models, not only DSLNT,^122^ but also 6'‐SL and 2'‐FL,^45^, ^123^, ^124^ showed some protection against the severity of NEC. Although a mechanism of action has yet to be established for DSLNT, 2'‐FL appears to ameliorate NEC symptoms through the modulation of endothelial nitric oxide synthase (eNOS) leading to increased gut perfusion.^123^ Interestingly, 2'‐FL was previously shown to improve vascularisation in another model system.^125^ Additionally, in mouse and piglet models of NEC, both 2'‐FL and 6'‐SL reduce clinical measures of NEC and inflammation, partly through the inhibition of TLR‐4 signalling, which is implicated in the onset of NEC.^45^ From *in silico* modelling, both 2'‐FL and 6'‐SL were predicted to dock to TLR‐4, thus inhibiting signalling. In other preterm pig models for NEC, 2'‐FL alone or in combination with other HMOs including 6'‐SL did not lead to significant reduction in NEC symptoms.^126^ Of note, in one study a blend of > 20 HMOs including DSLNT was tested, but did not reduce NEC symptoms in a preterm pig model.^126^ Although clinical observations have not shown associations between 2'‐FL or 6'‐SL with NEC, higher DSLNT concentrations in breast milk were found to be associated with, and to be a good predictor for, a lower risk of NEC in two independent preterm infant cohorts,^127^, ^128^ although this association was not confirmed in another smaller study.^129^ This latter study also reported a link between low HMO diversity in breast milk and NEC.^129^ Although possible explanations for the association between DSLNT and NEC remain elusive, a recent study^128^ suggests that DSLNT intake may be associated with a more age‐appropriate microbiome progression.^128^ Clearly, further research using an interventional design is needed to establish a causal link between DSLNT and NEC risk. It would also be worthwhile to investigate whether the same benefits of DSLNT on reduced NEC risk are observed with donor breast milk as with mothers’ own milk reported in the current studies. This will allow us to better understand whether additional maternal factors need to be considered in combination with the HMOs to understand their possible physiological role.

The role of breastfeeding in relation to risks of developing allergic diseases is ambiguous^9^, ^130^, ^131^, ^132^ possibly partly because of the large variability in breast milk composition. As iterated before, HMO composition in breast milk is highly variable and has profound effects on the neonatal microbiome development, which itself is related to sensitisation and development of allergic manifestations.^133^, ^134^, ^135^ Hence, several studies have investigated possible associations between the HMO composition of human milk and allergic manifestations in breastfed infants.

In a Finnish cohort (*n* = 266), infants with a hereditary risk of developing allergies and born by Caesarian section had an earlier onset of immunogloulin E‐associated eczema when breastfed by secretor‐negative mothers compared to those fed by secretor‐positive mothers.^136^ The infants fed secretor‐negative breastmilk also showed a more pronounced delay in establishing a bifidobacterial‐dominated microbiome at 3 months of age compared to fed with secretor‐positive breast milk.^137^ Among the affected bifidobacteria, specifically *B. breve* has been associated in independent studies to reduce the risk of pediatric eczema.^138^, ^139^ As summarised in a recent systematic review,^139^ observational data indicate that lower Bifidobacteriaceae abundance in infancy is associated with a higher risk of eczema, especially in infants with family history of atopy. However, the underlying mechanisms and the individual *Bifidobacterium* species involved remain unknown.

A small case–control study of 20 mother‐infant pairs from a larger birth cohort in Sweden found no association between the concentrations of nine neutral HMOs and risk of developing allergic disease up to 18 months of age.^140^ In another case–control study (*n* = 39 and 41), several individual HMOs (LNFP III, LNFP I, 6'‐SL, DSLNT) were observed to be lower in breast milk fed to infants with cow milk protein allergy, with LNFP III showing the strongest signal^141^ compared to non‐allergic infants. Of these, not only 6'‐SL, but also 2'‐FL provided some protection against development of a food allergy compared to lactose in an ovalbumin food allergy animal model.^142^ The mechanism for this effect was probably partly related to mast cell stabilisation leading to less histamine release. For LNFP III, further mechanistic insight may be gained based on its immune modulatory functions.^143^, ^144^

Another large clinical observation study (*n* = 421 mother–infant dyads) did not observe any association between individual HMOs and food sensitisation.^145^ Instead, out of the 19 measured HMOs, a specific profile classified using projection on latent structures‐discriminant analysis was found to be related to lower risk for food sensitisation. This profile could be characterised by relative higher concentrations of FDSLNH, LNFPII, LNnT, LNFPI, LSTc and FLNH, and relatively lower concentrations of LNH, LNT, 2'‐FL and DSLNH. Similarly, in another birth cohort (*n* = 285) of infants at risk of allergies, specific HMO profiles classified by latent class analysis (LCA) were reported to be associated with allergies up to 18 years of age.^146^ Although the approach to classify HMOs in profiles is promising and deserves to be extended to other breast milk components, its interpretability can be challenging. For example, Lodge et al.^146^ used LCA, a method that works with binary ‘yes/no’ data. To transform the HMO concentrations into a ‘yes/no’ signal, HMO was considered as ‘yes’ when above the median and as ‘no’ when below it.

To understand if and how HMOs modulate sensitisation and allergy risk in breastfed infants, large well‐controlled cohort studies with nested case‐control analysis are needed. To help interpretability, it may be useful to include HMO classifications with large differences between HMO concentrations. For example, profiles determined by maternal FUT2 and FUT3 genotypes or classifications considering highest and lowest quartile comparison. In addition to HMOs, other known immune active breast milk components such as transforming growth factor β should also be considered. The developing gut microbiome may also strongly affect expected functions of HMOs and should therefore be part of such investigations. Information may be gained by machine learning approaches to better understand whether symptoms or sensitisation can be explained by a combination of input features such as infant and maternal parameters, environmental factors, HMOs and infant microbiome data.

To date, no randomised controlled intervention trial has investigated the role of HMOs in the prevention of sensitisation and allergic diseases. Only one intervention trial assessed plasma cytokine profiles as a proxy for immune maturation in a subgroup analysis of infants fed formula supplemented with 2'‐FL at two concentrations and in combination with GOS, against only GOS in the control formula.^147^ Infants who received 2'‐FL in the formula showed similar basal and stimulated plasma cytokine profiles compared to the profiles in breastfed infants, but not those who received formula with GOS alone.

### HMOs and infectious illnesses

HMOs are largely undigested by the infant digestive enzymes. This observation, together with the recognition that the HMOs resemble mucous and cell surface glycans, triggered the hypothesis and concept that HMOs may serve as soluble ligands for pathogens and their toxins, as these often first attach via glycan ligands to epithelial cells.^148^, ^149^ Today, many different gastrointestinal and respiratory tract viral and bacterial pathogens have been shown to either bind to specific HMOs, or specific HMOs were shown to block adhesion of specific pathogens.^150^ Interestingly, specific HMOs, including LNT for example, were reported to have antibacterial activity by interfering with biofilm formation, cell wall synthesis and function of opportunistic pathogens such as *Streptococcus agalactiae* (Group B *Streptococcus*, GBS), *Staphylococcus aureus* and *Acinetobacter baumannii*.^77^, ^151^ Such HMO fragilised bacteria were shown to be more sensitive to antibiotic treatment.^77^, ^80^, ^152^ Interestingly, Chambers et al.^80^ reported increased 12,13‐DiHOME production in GBS treated with HMOs. Possibly, *in vivo* this may trigger an increased effector immune response for pathogen clearance.

In clinical observation studies, primarily the alpha 1,2‐linked fucosylated‐HMOs were associated with protection from infectious illnesses. In a pioneering study with 93 breastfed infants and their mothers from Mexico, Morrow et al.^153^ observed fewer cases of enteropathogenic induced diarrhea in infants of mothers expressing higher amounts of alpha 1,2‐linked fucosylated‐HMOs. Notably, this was observed for diarrhea caused by calicivirus, including norovirus, and for *Campylobacter*, against which 2'‐FL was specifically suggested to be protective. In two different mouse models, 2'‐FL was shown to reduce *Campylobacter jejuni* load and clinical symptoms. Moreover, *in vitro* adhesion to model cells was strongly reduced by 2'‐FL.^154^, ^155^ For specific norovirus strains, binding of 2'‐FL and also 3‐FL was shown to lead to reduced adhesion to their blood group antigen ligands *in vitro*.^156^, ^157^ Earlier work showed inhibition of norovirus particles by secretor‐positive milk, but not secretor‐negative milk indicating alpha 1,2‐linked fucosylated‐HMOs might be involved.^158^ However, HMOs seem not to have been involved. Rather, the alpha 1,2‐linked fucosylated‐glycans on milk mucins and lipase were found to inhibit norovirus adhesion.^158^ Alpha 1,2‐linked fucosylated‐HMOs were associated with reduced diarrhea and morbidity in independent cohorts in Africa.^107^, ^159^ Additionally, in another small study, the HMO LNFP II that is FUT3 dependent was associated with reduced gastrointestinal and respiratory illnesses in early infancy.^160^

From a molecular point of view, fragilising, blocking and deviating pathogens from adhering to their cognate cell surface ligands comprise plausible mechanisms of action for HMOs. These add a line of innate protective functions to the colonisation resistance brought about by an appropriately developing gut microbiome. In relation to respiratory pathogens, an additional effect is expected through gut microbial metabolites as elegantly demonstrated in basic research models that show protection from respiratory viral infections through immune active microbial metabolites.^86^, ^161^ Together, an intricate interplay between different breast milk glycan structures, including free HMOs, the gut microbiome and specific pathogens, is expected.

Not only infants, but also adults, who are genetic non‐secretors, are often at a lower risk of diarrheal and respiratory infections caused by specific pathogens.^162^, ^163^ This is an important confounding factor especially when investigating associations between HMOs that strongly depend on maternal secretor status and infectious illnesses in breastfed infants. Although breastfeeding reduces the risk of diarrhea, the exact HMO composition of breast milk as determined by maternal secretor status might not have a large impact on this protective effect. This indicates that a certain functional redundancy may exist within the diversity of HMOs in mother's milk. Mechanistically, this can be exemplified by the similar effects of 2'‐FL and 3‐FL on *B. longum* subsp *infantis*,^164^ and of 2'‐FL and 6'‐SL on models of NEC and allergic disease.^45^, ^142^

To date, only few individual manufactured HMOs, 2'‐FL alone or in combination with LNnT, have been tested in randomised controlled intervention trials in formula fed infants^109^, ^110^, ^111^, ^112^, ^113^, ^114^ and children.^164^ All trials in infants investigated growth as the primary safety objective and also investigated infectious morbidity, not only as part of the reporting of adverse events, but also with an *a priori* hypothesis to investigate whether HMOs reduce infectious illnesses. For 2'‐FL alone at 0.25 g L^–1^, Storm et al.^111^ reported a trend for a lower number of the overall infection related adverse events compared to controls. On the other hand, Marriage et al.^110^, ^147^ observed a higher incidence rate for reported adverse events related to overall infections in the control group of infants (GOS alone) and infants fed formula with 2'‐FL at 1 g L^–1^ with GOS compared to infants fed the lower dose of 2'‐FL at 0.2 g L^–1^ with GOS. In a cohort of healthy children aged 1–2.5 years, 2'‐FL at 3 g L^–1^ provided in two portions of 200 mL per day over a 6‐month period did not change the incidence of upper respiratory tract, nor gastrointestinal tract infections.^165^ Rather, and somewhat surprisingly, a slight increase in duration of upper respiratory tract infections was observed. Infants fed formula with the two HMOs 2'‐FL and LNnT experienced significantly fewer reported lower respiratory tract infections up to 1 year of age.^109^ In the same trial, infants fed the formula with 2'‐FL and LNnT also required significantly less antipyretics and prescribed antibiotics compared to control formula fed infants. Although these observations were based on an a priori hypothesis, they were part of the analysis of exploratory outcome measures in the trial. The observation of a lower risk of respiratory infections and lower need for antibiotics with 2'‐FL and LNnT supplementation is further supported by recent studies linking a microbiome community structure highly dominated by *Bifidobacterium* species at 3 months of age with a reduced requirement for antibiotics.^47^ Additionally, metabolites such as acetate, derived from HMO stimulated *Bifidobacterium* metabolic activity, could also contribute to a lower risk of respiratory tract infections.^166^ For example, in basic research models, acetate was shown to be protective against gastrointestinal pathogenic *Escherichia coli* ^85^ and respiratory syncytial virus through a type of interferon mediated pathway.^86^ Similarly, in cow milk protein allergic infants fed extensively hydrolysed formula feeding with the same 2'‐FL and LNnT, there was a trend for lower respiratory tract infections and antibiotic use in supplemented vs. control fed infants although this difference did not reach statistical significance because of the limited sample size.^113^

### HMOs and cognitive development

The brain is highly sialylated and many developmental and functional processes in the brain depend on sialic acid bound to proteins and glycolipids (i.e., gangliosides). As a result of the high sialic acid demand during early development and the high sialic acid content in breast milk, primarily in the form of HMOs, sialic acid is considered an important conditional nutrient in early life.^167^, ^168^, ^169^ Studies in animal models suggest that most dietary sialic acid is largely catabolised to pyruvate and GlcNAc and is not used directly as sialic acid,^26^, ^170^, ^171^ whereas some is reused directly through a salvage pathway as shown by the uptake and incorporation of the non‐human sialic acid NeuGc.^172^ Although the mechanisms are not fully established, these studies have led to the hypothesis that sialylated HMOs play a role in brain and cognitive development.

Today, numerous basic research models indeed support that sialyllactoses affect brain and cognitive development. In preterm pigs, a bovine milk preparation with sialyllactoses improved cognitive performance and upregulated hippocampal genes of sialic acid metabolism, ganglioside biosynthesis and myelination, whereas the concentration of hippocampal sialic acid was not affected.^173^ In neonatal pig studies, sialyllactose supplementation increased ganglioside bound sialic acid in the corpus callosum and cerebellum,^174^ affected sialic acid profiles in additional brain regions such as the prefrontal cortex as well as the hippocampus,^175^ and affected metabolic signatures including neurotransmitters.^176^ However, such changes did not translate into improved recognition memory or sleeping patterns.^177^ In different rodent models, 3'‐SL and 6'‐SL were found to improve learning and memory using different testing paradigms and models.^28^, ^178^, ^179^ Using a cross‐feeding model with dams genetically unable to synthesise 6'‐SL in their milks, wild‐type animals fed 6'‐SL deficient milk showed long lasting deficits in prefrontal cortex mediated executive functions.^28^ Analysis of early life brain, plasma and gut microbiota hinted to affected serotoninergic pathways, linking the gut and brain, as well as neurochemical and neuroanatomical adjustments in the brain. In another rodent experiment, using social disruption as a stressor, both 3'‐SL and 6'‐SL feeding prevented stress‐induced dysbiosis and anxiety such as behavior indicating that, at least in part, these HMOs may act via the microbiome involving the gut–brain axis pathways.^180^

Several studies have investigated the role of sialyllactoses in breastfed infants. In a cohort of 99 infant–mother dyads, a higher breast milk 3'‐SL concentration was associated with higher scores for expressive and receptive language development. However, this association was seen only in infants who were fed breast milk that contained the HMO A‐tetrasaccharide (only produced by mothers with blood group A) but not in infants fed breast milk without this HMO.^181^ In Malawian breastfed infants receiving FUT2 positive milk (*n* = 485), total sialylated HMOs, especially the concentration of total fucosylated HMOs, was positively associated with language development, whereas the non‐sialylated and non‐fucosylated HMOs structures showed an inverse relation.^96^ In a pilot study, breast milk 6'‐SL amounts at 1 month of age correlated positively with the composite cognitive score at 18 months of age (*n* = 76).^182^

Similar to sialyllactoses, 2'‐FL is also reported in numerous basic research models to help improve cognitive development. The first studies found that brain exposure to 2'‐FL, but not fucose or 3‐FL improved hippocampal long‐term potentiation.^183^, ^184^ Interestingly, direct effects of 2'‐FL and 3‐FL on enteric neuronal functions were also postulated from findings with an *ex vivo* model on gut contractility. Both 2'‐FL and 3‐FL, but not sialyllactoses, LNnT or GOS, had an immediate effect on colonic motor contractions, indicating that this effect is probably not driven by the gut microbiome.^185^ Furthermore, additional *ex vivo* tests with animals that were subjected to restraint stress showed that 2'‐FL alleviated stress‐induced gut dysmotility.^186^ Several recent studies tested 2'‐FL feeding in rodent models to assess cognitive abilities and its possible mode of action.^187^, ^188^ The long lasting and improved learning and memory outcomes with 2'‐FL feeding were related to effects on hippocampal memory related gene expression and long‐term potentiation. In additional studies, the effect of 2'‐FL was shown to be mediated via the vagus nerve^188^ and not by direct uptake of 2'‐FL or derived fucose into the brain.^189^, ^190^ Rather, for any uptake, microbial cleavage of 2'‐FL is necessary. There are relatively few data in humans but, in one study of breastfed infants, greater 2'‐FL intake at 1 month of age predicted better infant cognitive development at 24 months of age.^191^

Although infant cognitive development is affected by multiple environmental and nutrition factors, emerging data raise the possibility that HMOs make an important contribution and could partly help to explain some of the cognitive advantages of breastfeeding compared to formula feeding. Although the exact nature and mechanisms are not fully established, gut–brain communication processes involving gut microbiome metabolites are likely to be important. Well controlled and designed intervention trials with specific HMOs will be required to establish a causal link in this emerging field.

## CONCLUSIONS AND OUTLOOK

From a structural perspective, HMOs represent numerous structural features that are generally present on mucosal and cell surface glycans and play important modulatory roles in cell–cell and host–microbe interactions. From a physiological perspective, HMOs show many structure function‐specific activities, only observed with specific HMO species and not generally seen with unrelated glycans that are often used as prebiotics. However, there is redundancy of some functions between different HMO species, possibly acting as safeguard mechanisms for some of their important roles.

Although human milk is particularly rich in amounts and structural diversity of HMOs, some oligosaccharides are common across the animal milks. For example, 3'‐SL appears to be quite universally present in animal and human milks, suggesting universal and important functions across mammals.

Recent progress in manufacturing of individual HMOs has triggered a revival of research and great interest in the application of HMOs as seen by the exponential increase in published studies. Together, the cumulative evidence indicates that HMOs are a meaningful and important component of human milk, the optimal nutrition for early life. Increasing evidence also suggests that specific HMOs help establish immune competence, both local and systemically, partly through their effect on the metabolite activity of specific microbes such as specific *Bifidobacterium* species. HMOs may also participate in a gut–brain connection, thereby modulating brain and cognitive development. As expected from many biological processes, HMOs work in concert with other bioactive components and additionally act via different mechanisms that converge to specific functions.

Although we have started to accumulate clear evidence on the benefits of specific individual HMOs and blends thereof from randomised controlled trials, observational studies in breastfed infants have added to our knowledge and evidence supporting the importance of HMOs in early life. However, why human milk contains such diverse HMOs and what are the key drivers besides genetic polymorphism and time of lactation that explain the high variability in amounts of some HMOs remain key questions. To what extent the microbes and milk composition provided by mothers to their infants is personalised also remains an intriguing question for future research.

## CONFLICT OF INTERESTS

Norbert Sprenger, Hanne L. P. Tytgat, Aristea Binia and Sean Austin are employees of Société des Produits Nestlé, Switzerland. Atul Singhal has no conflict of interst regarding this work, but previously received research funding from Nestlé and Abbott Plc, as well as honoraria to give lectures and attend advisory boards for Nestlé Nutrition Institute, Danone, Wyeth Nutrition, Reckitt, Phillips, Abbott Nutrition and several academic institutions.

## ETHICS STATEMENT

Not applicable.

## AUTHOR CONTRIBUTIONS

Norbert Sprenger conceptualised and drafted the manuscript. Atul Singhal, Sean Austin and Atul Singhal completed the writing of the manuscript. Norbert Sprenger and Hanne L. P. Tytgat prepared the figures. Norbert Sprenger and Sean Austin prepared the tables. All authors reviewed and approved the final version of the manuscript submitted for publication.

## TRANSPARENCY DECLARATION

The peer review history for this article is available at https://publons.com/publon/10.1111/jhn.12990.

## Data Availability

Data sharing not applicable to this article as no datasets were generated or analysed during the current study.
